# The relation between survival and expression of HER1 and HER2 depends on the expression of HER3 and HER4: a study in bladder cancer patients

**DOI:** 10.1038/sj.bjc.6603154

**Published:** 2006-05-09

**Authors:** A A Memon, B S Sorensen, P Meldgaard, L Fokdal, T Thykjaer, E Nexo

**Affiliations:** 1 Department of Clinical Biochemistry, NBG, AS, Aarhus University Hospital, 8000 Aarhus C, Norrebrogade 44, Denmark; 2 Department of Oncology, NBG, AS, Aarhus University Hospital, Norrebrogade 44, 8000 Aarhus C, Denmark; 3 Department of Clinical Biochemistry, Skejby Hospital, 8200 Aarhus N, Norrebrogade 44, Denmark

**Keywords:** ErbB, HER, EGFR, epidermal growth factor, bladder cancer

## Abstract

Increased expression of the epidermal growth factor (EGF) receptors, HER1 and HER2 are related to poor prognosis in most cancers studied. Recently, a high expression of the two remaining receptors of the EGF system, HER3 and HER4 has been related to a favourable prognosis. However, prognostic significance of HER1 and HER2 receptors in bladder cancer is controversial and the effect of the expression of different combinations of these receptors on patient survival is not well understood. Therefore, we examined the mRNA expression of all four EGF receptors with real-time polymerase chain reaction in biopsies from 88 patients with bladder cancer, where the survival was followed for a median of 38.5 months (range 1–117 months). Expression of HER1 and HER2 alone showed no correlation with survival. However, a high expression of HER1 together with high expression of HER3 and HER4 correlated to a better prognosis compared to the high expression of HER1 together with low expression of HER3 and HER4 (*P*=0.0006). Also, a significantly longer survival was observed in patients expressing high HER2 when coexpressed with high HER3 and HER4, as compared to the survival in patients with tumours expressing high HER2 but low HER3 and HER4 (*P*=0.0005). Our results suggest that the final outcome of patients with high HER1- and HER2-expressing tumours depends on the expression of HER3 and HER4.

The epidermal growth factor (EGF) family of receptor tyrosine kinases comprises four members: HER1 (EGF receptor 1 Human EGF Receptor/ErbB1), HER2 (neu/ErbB2), HER3 (ErbB3) and HER4 (ErbB4) (Yarden and Sliwkowski, 2001). Epidermal growth factor receptors are commonly active in a dimeric form and interaction between different EGF receptor pairs represents a mechanism for signal diversification and amplification (Olayioye *et al*, 2000; Yarden and Sliwkowski, 2001). Various dimeric pairs depend on the concentration of receptors, the concentration of particular ligands and the affinity of the receptors towards each other (Pinkas-Kramarski *et al*, 1996; Tzahar *et al*, 1997). Ligands binding the EGF family receptors induce receptor homo- or heterodimerisation, which involves an array of a number of homodimeric and heterodimeric combinations (Burden and Yarden, 1997). The ligands for these receptors consist of approximately 20 different proteins (including isoforms) encoded by at least 10 different genes. The numerous EGF family-specific ligands include EGF and five other ligands able to bind to HER1. Whereas heregulins are the ligands for HER3 and HER4. No ligand for HER2 has been identified and HER3 lacks intrinsic tyrosine kinase activity (Guy *et al*, 1994). Thus, the normal mechanism by which HER2 or HER3 can signal is through heterodimerisation with other EGF receptors.

Epidermal growth factor receptors, particularly HER1 and HER2, are commonly deregulated in certain forms of human cancer including bladder cancer. It has been shown that HER1 and HER2 expressions are involved in poor prognosis (Garcia *et al*, 2003; Nielsen *et al*, 2004; Tovey *et al*, 2004). Despite the numerous studies devoted to the issue of clinical significance of HER1 and HER2, the role of these receptors is still controversial in bladder cancer.

The role of the HER3 and HER4 receptors is not well understood. However, growing consensus has emerged about the role of HER4, and the few clinical studies carried out so far have suggested that its expression is associated with favourable prognosis (Thybusch-Bernhardt *et al*, 2001; Suo *et al*, 2002; Rotterud *et al*, 2005). We have shown that HER3 and HER4 expression correlated with a better prognosis in bladder cancer patients (Memon *et al*, 2004). More recent reports also suggest that unlike HER1 and HER2, expression of HER3 and HER4 was not found in more aggressive metastatic squamous cell carcinomas of the oral cavity (Ekberg *et al*, 2005). The same was reported in meningiomas (Andersson *et al*, 2004). In addition, presence of HER4 in HER2-expressing tumours reduced the recurrence in breast cancer patients, further supporting the distinct roles for EGF family receptors (Barnes *et al*, 2005).

It has been demonstrated that EGF receptor heterodimers are more potent in signal transduction than homodimers. Heterodimers provide additional phosphotyrosine residues for the recruitment of effector proteins, and induce distinct patterns of receptor phosphrylation and downstream signalling. This suggests that the outcome of activation of the EGF system depends on the interaction between the EGF receptors.

The prognostic significance of all four receptors in different combinations remains unclear. Knowledge about this is important in order to design the best cancer treatment directed towards these receptors. Today, drugs directed towards HER1 (e.g. iressa/gefitinib) and HER2 (e.g. trastuzumab) (Diermeier *et al*, 2005) are already in clinical use and strategies involving inhibition of all four receptors are under evaluation. Therefore, it is important that all four EGF receptors should be analysed in the same group of patients before drawing any conclusion about the prognostic significance of these receptors. Lack to do so could be one of the reasons for the conflicting data about the role of HER1 and HER2 as prognostic markers.

Epidermal growth factor family gene expression can reliably be studied at the mRNA level and based on a number of studies, the expression is likely to reflect the presence of the corresponding protein (Knowlden *et al*, 1998; Walker and Dearing, 1999; Suo *et al*, 2002; Junttila *et al*, 2003).

The present study was undertaken in order to examine the pattern of expression of HER1-4 in bladder cancer biopsies from 88 patients in relation to survival of the patients. We report that a high expression of HER3 and/or HER4 protects the patients from the consequences of a high expression of HER1 and/or HER2.

## MATERIALS AND METHODS

### Patients

Eighty-eight patients with primary bladder cancer were included. Biopsies were obtained by transurethral tumour resection and alliqotes were frozen immediately. Samples for histological examination were removed before freezing and analysed independent of the further analysis of RNA. Tumour stage was assigned according to the Union Internationale Contre le Cancer Tumour-Node-Metastasis system (Spiessl *et al*, 2003). Grading was performed in accordance with methods described by Bergkvist *et al* (1965). The relationship between the distribution of tumour stage, age and sex is summarised in Table 1. At the time of inclusion, 18 patients had received treatment in the form of radical radiotherapy, chemotherapy or intravesical therapy with bacillus Calmette-Guerin. The follow-up period was from the date of biopsy to the day of death or to July 2005. Patients were censored if they were alive at the time of analysis (July 2005). The median follow-up was 38.5 months (range 1–117 months). The regional committee of Scientific Ethics, Aarhus approved the study, and the procedures were followed in accordance with the Helsinki Declaration.

### Preparation of total RNA

RNA preparation was performed as described before (Memon *et al*, 2004). Briefly, tumour samples were immediately placed in a denaturing solution (4 mol l^−1^ guanidine thiocyanate, 25 mmol l^−1^ sodium citrates (PH7), 0.5% sarkosyl and 0.1 mmol l^−1^ 2-mercaptoethanol) and stored at −80°C. A frozen biopsy (<20 mg) was homogenised by a Heidolph Diax 600 mixer. Total RNA was extracted from tissues according to a slightly modified method of Chomczynski and Sacchi (1987).

### Real-time reverse transcription–polymerase chain reaction quantification of mRNA

Quantification of mRNA was performed by real-time reverse transcription–polymerase chain reaction on the Lightcycler instrument (Roche, Germany) as described before (Memon *et al*, 2004). Briefly, cDNA was generated in a reverse transcription reaction, where 1 *μ*g RNA was mixed with 2.5 units AMW reverse transcriptase (Applied Biosytems, Foster city, USA) in a reaction mixture containing 10 mM Tris-HCl (pH 8.3), 1 U *μ*l^−1^ RNase inhibitor, 1 mmol l^−1^ deoxyribonucleoside triphosphate (dATP, dTTP, dGTP and dCTP), 2.5 *μ*mol l^−1^ 16 mer d(T)_16_ primer, 50 mmol l^−1^ KCl, 6.25 mmol l^−1^ MgCl_2_ in a total volume of 20 *μ*l (all reagents from Applied Biosystem). The reactions were incubated in a perkin Elmer 9700 thermocycler for 90 s at 94°C followed by 30 min at 42°C and finally at 94°C for 1 min. Real-time polymerase chain reaction (PCR) was performed with the Lightcycler Sybr Green I quantification kit (Roche) in a total volume of 10 *μ*l in LC glass capillaries (Roche). The HER1 primers: 5′-AGAGGAGAACTGCCAGAA-3′ (sense) and 5′-GTAGCATTTATGGAGAGTG-3′ (antisense); HER2 primers: 5′-CCAGGACCTGCTGAACTGGT-3′ (sense) and 5′-GTACGAGCCGCACATCC-3′ (antisense) give rise to a 454 and 272 bp PCR product, respectively. HER3 and HER4 quantifications were previously determined by same method by amplifying a 365 and 265 bp fragment, respectively (Memon *et al*, 2004). Beta-actin mRNA was used as an endogenous RNA control, which has been used as control gene in various studies on bladder cancer (Vageli *et al*, 1996; Chiu *et al*, 2002) as well as on breast cancer (Agudo *et al*, 2004). Specificity was verified by the size of the PCR product on agarose gel electrophoresis and nucleotide sequencing using a 310 genetic analyser (Applied Biosystems). A calibration curve and positive and negative controls were included in each run. The results are presented based on a calibration curve of mRNA. The calibration curve was composed of serial dilutions of a pool of mRNA isolated from; HCV cells for measurement of HER1 and HER2 whereas HEC and KLE cells were used for HER3 and HER4, respectively. Likewise, actin mRNA was analysed in the same samples employing mRNA isolated from HCV as calibrator. All the quantifications in this study are presented as ratio between the target gene and beta-actin. Quantifications were performed using Lighcycler Software Version 3 (Roche).

### Statistical analysis

Nonparametric tests were used throughout this study. Two-sided *P*-values <0.05 were considered to be significant. The *χ*^2^ test was used to compare the expression of the EGF family members with clinical stage, grade, tumour type and size of the tumour. Life table calculations were carried out using the Kaplan–Meier method. Comparison between the curves was carried out using log-rank test. (The software Graph Pad Prism (version 4) was used for statistical analysis.)

## RESULTS

### Pattern of EGF family receptor expression and correlation with histopathological parameters of bladder cancer

We report the mRNA expression of HER1 and HER2 in bladder cancer biopsies from 88 patients followed for a median of 38.5 months (range 1–117 months). HER3 and HER4 mRNA expressions have previously been determined on the same samples (Memon *et al*, 2004). HER1 was expressed in 96% (85 out of 88 patients) and HER2 in 98% (86 out of 88 patients) of the tumour samples. As previously reported (Memon *et al*, 2004), HER3 and HER4 expressions were found in 99 (87 out of 88 patients) and 63% (56 out of 88 patients) of the tumour samples, respectively. Median concentrations of each mRNA examined were selected as the cutoff point, dividing all patients into two groups, one with high expression (above median, denoted as (+)) and another with low expression (at or below median, denoted as (−)). High expressions of HER1 or HER2 when coexpressed with high HER3 and HER4 strongly correlated (*P*<0.05) with good prognostic indicators of bladder cancer (early stage of tumour (Ta–T1), low grade (Grade I/II) and papillary type of tumour). In contrast, tumours expressing only high HER1 or HER2 but low HER3 or HER4 correlated with poor prognostic parameters of bladder cancer (late stage of tumour (T2–T4), high-grade (Grade III/IV) and solid type of tumour). In relation to stages of the tumour, 77 and 79% of the patients coexpressing high levels of HER1, HER3 and HER4 (+HER1/+HER3/+HER4) (Table 2) or high levels of HER2, HER3 and HER4 (+HER2/+HER3/+HER4) (Table 3), respectively, correlated with early stage of the tumour (Ta–T1). Whereas, 100% of tumours overexpressing HER1 or HER2 with low expression of HER3 and HER4 (+HER1/−HER3/−HER4) and (+HER2/−HER3/−HER4) correlated with late stage (T2–T4) of the bladder cancer.

### Correlation with survival

**Figure 1:**
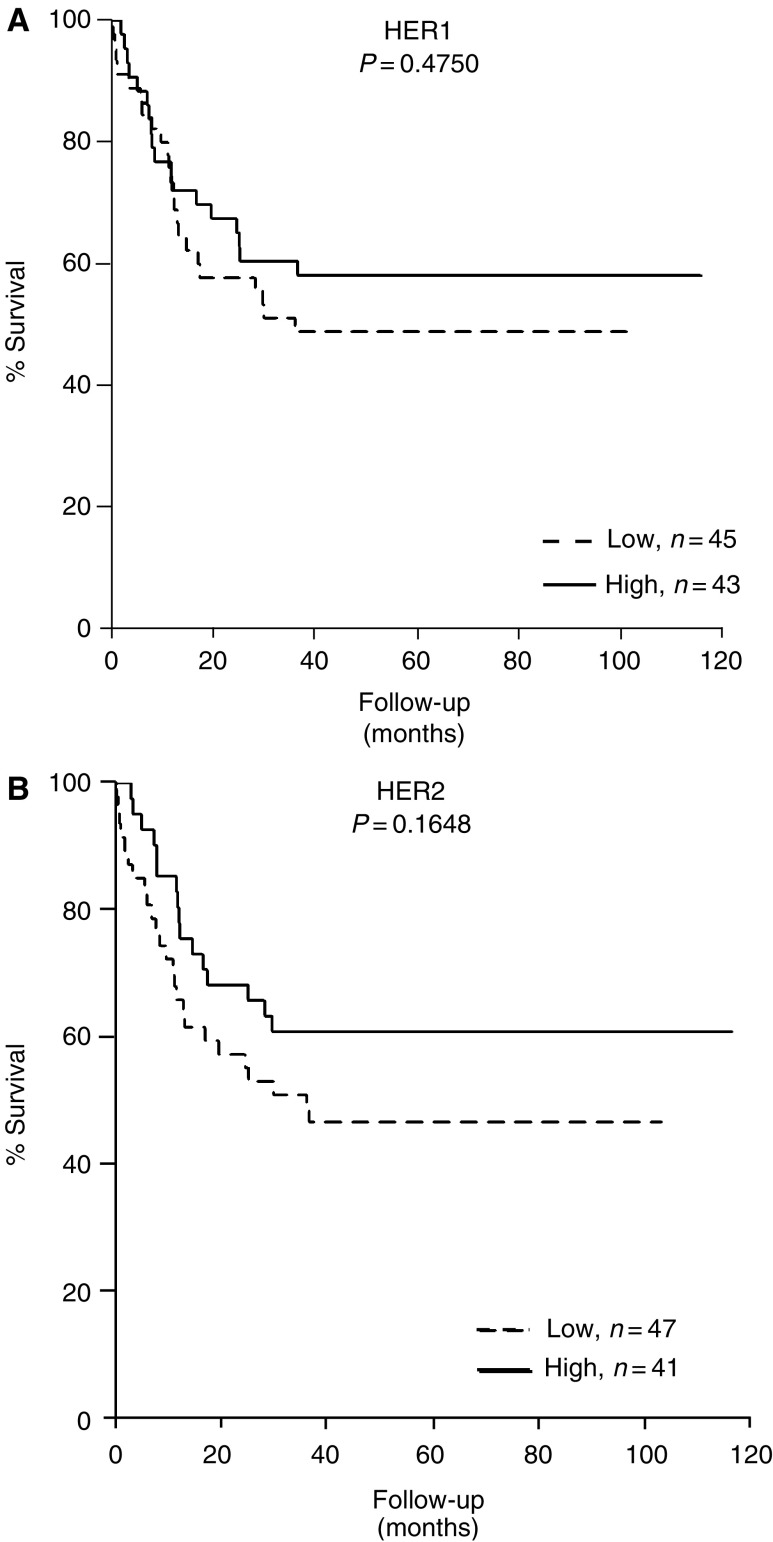
Kaplan–Meier overall survival analysis comparing patients with high (above median) and low (below median) HER1 (**A**) or HER2 (**B**) individual expression in 88 bladder cancer patients. *P*-value represents log-rank differences in survival between two groups.

**Figure 2:**
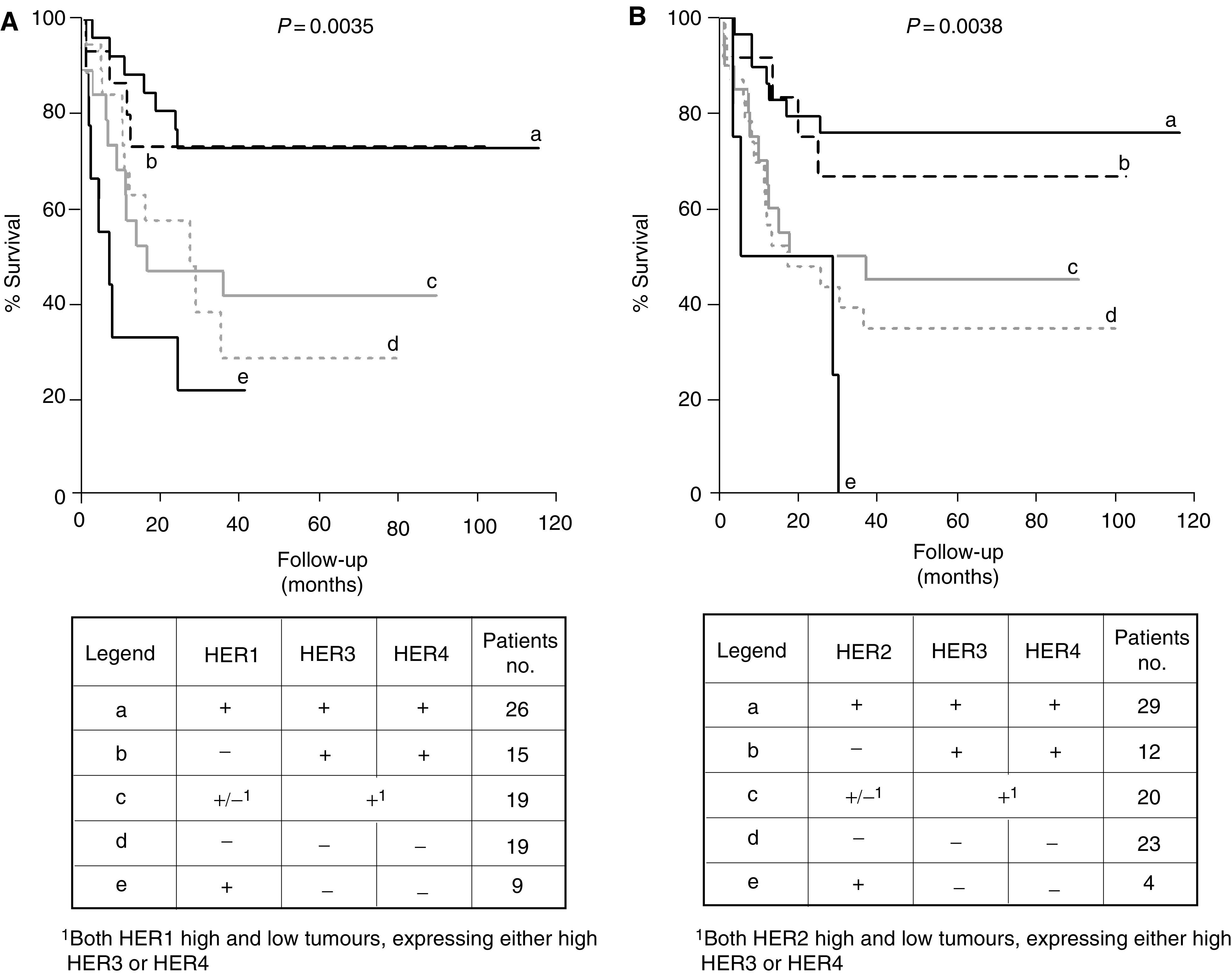
Kaplan–Meier survival curves demonstrating survival function of all EGF receptors in various combinations in 88 bladder cancer patients. HER1 (**A**) or HER2 (**B**) expressing tumours correlated to worst prognosis in the absence of both HER3 and HER4 expressions. Legend tables showing various combinations analysed. *P*-value represents log-rank difference in survival between all groups.

The median mRNA concentrations of each EGF receptors in all the bladder tumours were selected as the cutoff limit and patients were categorized into groups with high (above median) or low expression (below median). Kaplan–Meier survival curves were made to evaluate the impact of expression of HER1 and HER2 individually and in combination with HER3 and HER4. Patients were divided into the following groups. Group (a) patients expressing high HER1, HER3 and HER4, (b) patients expressing low HER1 with high HER3 and HER4, (c) patients expressing low all four receptors and (e) patients expressing high HER1 with low HER3 and HER4. There was a group of patients expressing high HER1 together with a high expression of either HER3 or HER4 and another group of patients expressing low HER1 together with a high expression of either HER3 or HER4, both groups were combined and shown as (d). The same grouping was performed for the HER2, HER3 and HER4 combinations (Figures 1 and 2present, legend tables).

Expression of either HER1 or HER2 (Figures 1A and B) or these two receptors in combination (data not shown) did not correlate with survival (*P*>0.05). However, patients having tumours expressing high HER1 together with high expressions of HER3 and HER4 had a better survival (Figure 2A) compared to the patients expressing high HER1 but low expression of HER3 and HER4 (*P*=0.0006) (Figure 2A, compare a with e). Also patients having tumours expressing high HER2 together with high expressions of HER3 and HER4 had a better survival (Figure 2B) than did the patients expressing high HER2 but low HER3 and HER4 (*P*=0.0005) (Figure 2B, compare a with e). It was also observed that the presence of high expression of either HER1 or HER2 did not affect the survival of high HER3- and HER4-expressing tumours (*P*>0.05). Patients with high HER3 and HER4 expression irrespective of the presence of high or low HER1 (Figure 2A, compare a and b) or HER2 (Figure 2B, compare a and b) showed comparable and favourable survival.

We also analysed the effect of high expression of HER3 (irrespective of the expression of HER4) together with high expression of HER1 or HER2. Likewise, the effect of high HER4 (irrespective of the HER3 expression) in combination with high expression of HER1 or HER2 was analysed. Our results showed that high expression of HER3 together with high HER1 or HER2 expression also correlated to better survival (data not shown). Similarly, high expression of HER4 together with high HER1 or HER2 expression also correlated to better survival (data not shown). However, the effect was less marked than observed for tumours where both HER3 and HER4 were coexpressed at a high level together with HER1 or HER2.

**Figure 3:**
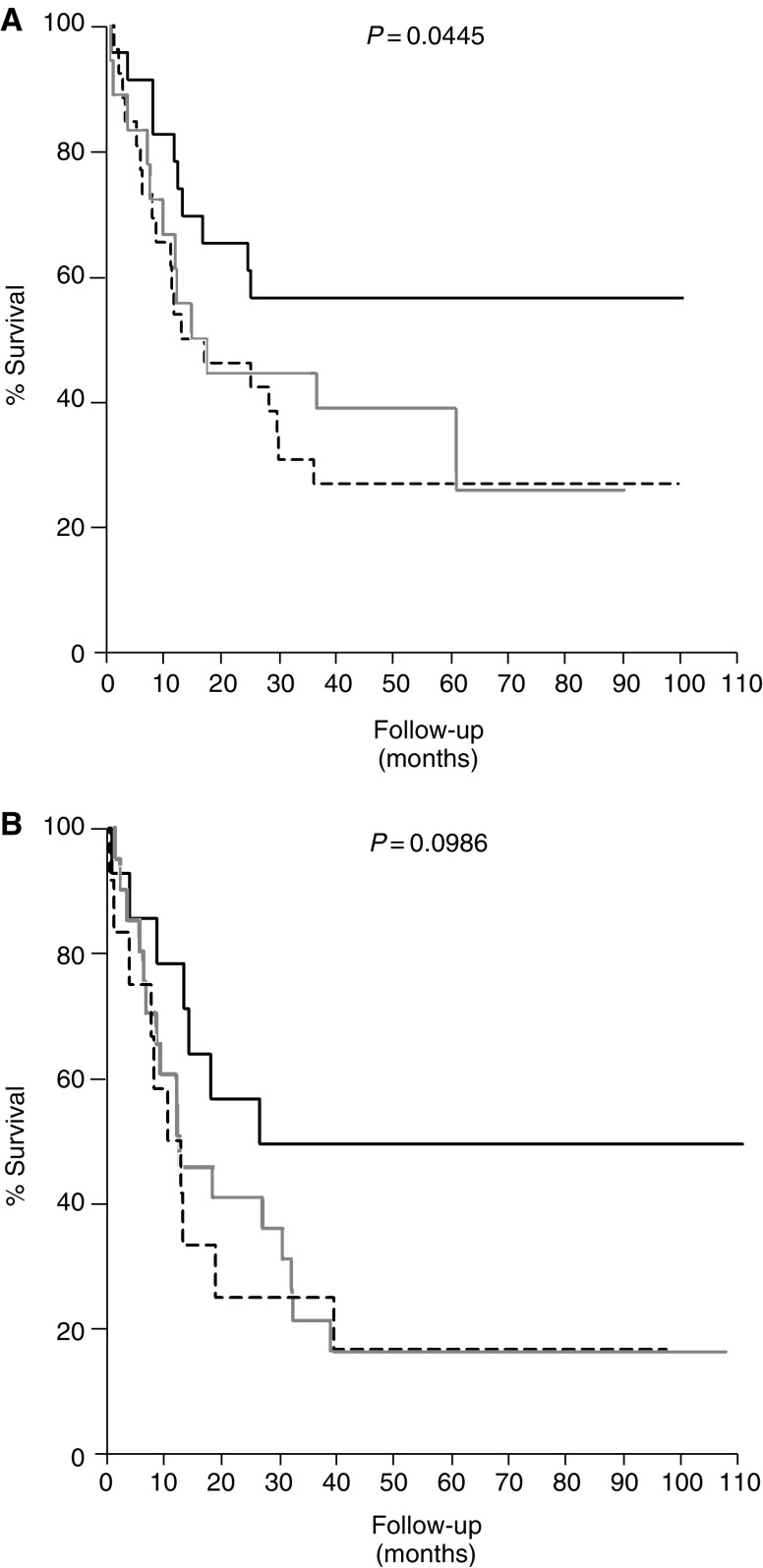
Kaplan–Meier curves showing survival analysis of coexpression of HER3 and HER4 in (**A**) patients with T1–T4 tumours (*n*=67) and (**B**) patients with solid and mixed type of bladder cancer (*n*=50). All patients were divided into three groups according to HER3 and HER4 coexpressions. Black line denotes patients with high HER3 and HER4 expressions (*n*=23), grey line denotes patients with low HER3 and HER4 expressions (*n*=26) and broken line denotes patients expressing either HER3 or HER4. *P*-value denotes log-rank differences in survival between high and low HER3 and HER4 expression.

We also analysed the relation between survival and HER3 and HER4 expressions in the subgroup of patients with invasive tumours (T1–T4). Our results show that expression of high HER3 and HER4 compared to low HER3 and HER4 correlated with better prognosis even in this highly invasive group of tumours (Figure 3A). In addition, we also analysed the survival function of HER3 and HER4 in the group of patients with solid and mixed type of tumours. In this group, we also found a trend showing that patients expressing high HER3 and HER4 had a better survival compared to the patients expressing low HER3 and HER4 (Figure 3B).

Finally, we examined whether treatment of patients before biopsies were taken, had any effect on our final conclusions, but we did not find this to be the case (data not shown).

## DISCUSSION

The EGF family receptors and its ligands are involved in cancer development and prognosis. Abnormal function of the members of the EGF family has been linked to bladder cancer prognosis. Several reports, based mainly on the expression of HER1 and HER2, demonstrate that the EGF family of receptors are involved in poor prognosis in various cancers including bladder cancer (Lonn *et al*, 1995; Arpino *et al*, 2004; Blackwell *et al*, 2004; Popov *et al*, 2004). In contrast to HER1 and HER2, our study on bladder tumours (Memon *et al*, 2004) and other studies on breast tumours (Abd El-Rehim *et al*, 2004) have suggested that increased expression of HER3 and HER4 is associated with improved survival. This is supported by data on cancer cells that demonstrate a ligand-dependent proapoptotic function of the HER4-expressing cells (Sartor *et al*, 2001). Furthermore, recent findings about HER4 expression in breast tumours also point towards a proapoptotic function of HER4 (Barnes *et al*, 2005). These studies demonstrate that different EGF receptors function differently, and that the expression pattern of the individual receptors may be of importance in determining the cellular outcome. However, very limited information is available where the expression of all four receptors has been analysed and related to the survival of bladder cancer patients.

Our results show that neither individual nor combined expression of HER1 and HER2 correlated with survival, in agreement with some of the previous reports on bladder cancer (Jimenez *et al*, 2001; Thogersen *et al*, 2001). However, conflicting reports have been published on the prognostic value of HER1 and HER2 receptors in bladder cancer. For example, HER1 and HER2 expressions were correlated with both good (Gandour-Edwards *et al*, 2002; Chakravarti *et al*, 2005) and poor (Kruger *et al*, 2002; Popov *et al*, 2004) prognosis in bladder cancer, whereas other suggest that there is either no or only limited prognostic significance of HER1 and HER2 expression in this disease (Mellon *et al*, 1996; Ravery *et al*, 1997).

*In vitro* studies suggest that cell lines expressing only one of the HER receptors were unable to form tumours in animals with the exception of HER1, which was weakly tumorigenic. Moreover, although unable to form tumours when expressed alone, HER2 was tumorigenic when expressed with HER1 or HER3, but not HER4. Of all combinations analysed, cells expressing both HER1 and HER2 were the most aggressive (Cohen *et al*, 1996). In addition, when HER2-expressing cells were transfected with HER4, antiproliferative and differentiation responses were observed (Sartor *et al*, 2001), suggesting that HER4 signalling has an opposite effect than HER2 signalling.

Based on these observations, we hypothesised that the final outcome of the bladder cancer patients may depend on the balance between the expressions of EGF receptors.

We demonstrate that a high expression of either HER1 or HER2 has no effect on survival when HER3 and HER4 are present. In contrast, high expressions of either HER1 or HER2, in tumours where HER3 and HER4 are low, result in significantly reduced survival. In our group of patients, we observed that 12 patients were expressing high concentrations of HER1 or HER2 with low HER3 and HER4, and 10 of these patients died during the course of follow-up (8 months median follow-up). In contrary, 34 patients expressing high HER1 or HER2 together with high HER3 and HER4 correlated with longer survival (44 months median follow-up), and only 10 out of 34 patients died during the follow-up period. This suggests that expression of HER1 and HER2 is involved in tumour progression and poor prognosis in the bladder tumours only when HER3 and HER4 are low. In keeping with our results, studies on breast cancer investigating the expression of the individual EGF receptors also show that increased HER3 and HER4 expression appears to be associated with better prognosis (Quinn *et al*, 1994; Knowlden *et al*, 1998). However, it should be noted that in another study, only HER4 was found to be associated with a better prognosis whereas HER1, HER2 and HER3 overexpression was associated with a poor outcome (Witton *et al*, 2003).

In this study, we are able to show that HER3 and HER4 expression can influence the effect of HER1 and HER2 on survival. These data emphasise that combined analysis of the expression of all four EGF receptors provide more accurate information on the tumour behaviour than analysis of the expression of the individual receptors. Therefore, one possibility for the conflicting data on the consequences of high HER1 and HER2 expression in bladder cancer patients published until now could be related to the function of HER3 or HER4 expression, which was ignored in most of the studies.

In conclusion, our results suggest that expression of different combinations of receptor can change the final outcome of the disease, and we suggest that the expression of HER3 and HER4 should be taken into account for future evaluation of the consequences of HER1 and HER2 expression in bladder cancer. This might also be of importance in identifying patients, which may benefit from the specific antitumoural drugs designed to target the EGF receptors.

## Figures and Tables

**Table 1:** Clinical data in 88 bladder cancer patients

**Variables**	**No. of patients**
*Sex*	
Male	69
Female	19
	
*Age (years)*	
Median	72
Range	53–88
	
*Stage*	
Ta	21
T1	18
T2–T4	49
	
*Grade*	
I/II	26
III/IV	62
	
*Tumour type*	
Papillary	38
Solid	37
Mixed	13
	
*Tumour size* (cm)	
<3	50
>3	38

**Table 2:** Correlation between combinations of HER1 and HER3/HER4 and clinicopathological parameters of bladder cancer

	**Receptor combinations**					
**HER1**	+	−	**+/−a**	−	+	
**HER3**	+	+	**+/−a**	−	−	
**HER4**	+	+	**+/−a**	−	−	***P* b**
All tumours	26^c^ (30.0)^d^	15 (17.0)	19 (22.0)	19 (22.0)	9 (10.0)	
*Stage*						
Ta–T1	20 (77.0)	10 (67.0)	2 (11.0)	5 (26.0)	0 (0.0)	0.0002
T2–T4	6 (23.0)	5 (33.0)	17 (89.0)	14 (74.0)	9 (100)	
						
*Grades*						
Grade I+II	17 (65.0)	5 (33.0)	2 (11.0)	2 (11.0)	0 (0.0)	0.0018
Grade III+IV	9 (35.0)	10 (67.0)	17 (89.0)	17 (17.0)	9 (100)	
						
*Size* (cm)						
<3	15 (58.0)	10 (67.0)	8 (42.0)	13 (68.0)	4 (44.0)	NS
>3	11 (42.0)	5 (33.0)	11 (58.0)	6 (32.0)	5(56.0)	
						
*Tumour type*						
Papillary	19 (73.0)	6 (40.0)	6 (32.0)	4 (21.0)	2 (22.0)	0.0042
Solid	3 (12.0)	6 (40.0)	7 (36.0)	14 (74.0)	7 (78.0)	
Mixed	4 (15.0)	3 (20.0)	6 (32.0)	1 (5.0)	0 (17.0)	

NS=not significant.

a Both high and low HER1 tumours expressing either high HER3 or high HER4.

b *χ*^2^ test, analysis was performed between combinations of +HER1/+HER3/+HER4, +HER3/+HER4 and +HER1 only expressing tumours.

c No. of patients.

d Percentage.

**Table 3:** Correlation between combinations of HER2 and HER3/HER4 and clinicopathological parameters of bladder cancer

	**Receptor combinations**					
**HER2**	+	−	**+/−a**	−	+	
**HER3**	+	+	**+/−a**	−	−	
**HER4**	+	+	**+/−a**	−	−	***P* b**
All tumours	29^c^ (33.0)^d^	12 (14.0)	20 (22.0)	23 (26.0)	4 (5.0)	
*Stage*						
Ta–T1	23 (79.0)	7 (58.0)	5 (25.0)	4 (17.0)	0 (0.0)	0.0054
T2–T4	6 (21.0)	5 (42.0)	15 (75.0)	19 (83.0)	4 (100)	
						
*Grades*						
Grade I+II	16 (55.0)	6 (50.0)	2 (11.0)	2 (9.0)	0 (0.0)	NS
Grade III+IV	13 (45.0)	6 (50.0)	18 (89.0)	21 (91.0)	4 (100)	
						
*Size* (cm)						
<3	21 (72.0)	4 (33.0)	9 (45.0)	14 (61.0)	2 (50.0)	NS
>3	8 (28.0)	8 (77.0)	11 (55.0)	9 (39.0)	2 (50.0)	
						
*Tumour type*						
Papillary	21 (73.0)	5 (42.0)	6 (30.0)	6 (26.0)	0 (0.0)	0.0017
Solid	3 (10.0)	6 (50.0)	8 (40.0)	16 (70.0)	4 (100)	
Mixed	5 (17.0)	1 (8.0)	6 (30.0)	1 (4.0)	0 (0.0)	

NS=not significant.

a Both high and low HER2 tumours expressing either high HER3 or high HER4.

b *χ*^2^ test, analysis was performed between combinations of +HER2/+HER3/+HER4, +HER3/+HER4 and +HER1 only tumours.

c No. of patients.

d Percentage.
